# Transmission of COVID-19 to Health Care Personnel During Exposures to a Hospitalized Patient — Solano County, California, February 2020

**DOI:** 10.15585/mmwr.mm6915e5

**Published:** 2020-04-17

**Authors:** Amy Heinzerling, Matthew J. Stuckey, Tara Scheuer, Kerui Xu, Kiran M. Perkins, Heather Resseger, Shelley Magill, Jennifer R. Verani, Seema Jain, Meileen Acosta, Erin Epson

**Affiliations:** ^1^California Department of Public Health; ^2^Epidemic Intelligence Service, CDC; ^3^CDC COVID-19 Response Team; ^4^Solano County Public Health, Fairfield, California; ^5^NorthBay Healthcare, Fairfield, California.

On February 26, 2020, the first U.S. case of community-acquired coronavirus disease 2019 (COVID-19) was confirmed in a patient hospitalized in Solano County, California (*1*). The patient was initially evaluated at hospital A on February 15; at that time, COVID-19 was not suspected, as the patient denied travel or contact with symptomatic persons. During a 4-day hospitalization, the patient was managed with standard precautions and underwent multiple aerosol-generating procedures (AGPs), including nebulizer treatments, bilevel positive airway pressure (BiPAP) ventilation, endotracheal intubation, and bronchoscopy. Several days after the patient’s transfer to hospital B, a real-time reverse transcription–polymerase chain reaction (real-time RT-PCR) test for SARS-CoV-2 returned positive. Among 121 hospital A health care personnel (HCP) who were exposed to the patient, 43 (35.5%) developed symptoms during the 14 days after exposure and were tested for SARS-CoV-2; three had positive test results and were among the first known cases of probable occupational transmission of SARS-CoV-2 to HCP in the United States. Little is known about specific risk factors for SARS-CoV-2 transmission in health care settings. To better characterize and compare exposures among HCP who did and did not develop COVID-19, standardized interviews were conducted with 37 hospital A HCP who were tested for SARS-CoV-2, including the three who had positive test results. Performing physical examinations and exposure to the patient during nebulizer treatments were more common among HCP with laboratory-confirmed COVID-19 than among those without COVID-19; HCP with COVID-19 also had exposures of longer duration to the patient. Because transmission-based precautions were not in use, no HCP wore personal protective equipment (PPE) recommended for COVID-19 patient care during contact with the index patient. Health care facilities should emphasize early recognition and isolation of patients with possible COVID-19 and use of recommended PPE to minimize unprotected, high-risk HCP exposures and protect the health care workforce.

HCP with potential exposures to the index patient at hospital A were identified through medical record review. Hospital and health department staff members contacted HCP for initial risk stratification and classified HCP into categories of high, medium, low, and no identifiable risk, according to CDC guidance.* HCP at high or medium risk were furloughed and actively monitored; those at low risk were asked to self-monitor for symptoms for 14 days from their last exposure.^†^ Nasopharyngeal and oropharyngeal specimens were collected once from HCP who developed symptoms consistent with COVID-19^§^ during their 14-day monitoring period, and specimens were tested for SARS-CoV-2 using real-time RT-PCR at the California Department of Public Health. Serologic testing and testing for other respiratory viruses was not performed.

The investigation team, including hospital, local and state health departments, and CDC staff members, attempted to contact all 43 tested HCP by phone to conducted interviews regarding index patient exposures using a standardized exposure assessment tool. Two-sided p-values were calculated using Fisher’s exact test for categorical variables and Wilcoxon rank-sum test for continuous variables; p-values <0.05 were considered statistically significant. Analyses were conducted using SAS (version 9.4; SAS Institute). The California Health and Human Services Agency’s Committee for the Protection of Human Subjects and CDC determined this investigation to be public health practice.

Hospital A identified 145 HCP with potential exposure to the index patient. After the initial interview, 24 (17%) HCP were classified as having no identifiable risk; the remaining 121 were classified as having high (14), medium (80), or low (27) risk. Over the course of their monitoring periods, 43 (36%) of these HCP became symptomatic and underwent testing for SARS-CoV-2, with a median of 10 days from last exposure to specimen collection (Table 1); SARS-CoV-2 was detected in three (7%) HCP. Thirty-seven of 43 (86%) HCP who were tested were interviewed, including all three HCP with positive test results.^¶^

**TABLE 1:** Demographic characteristics, exposure risk categories, and job titles of 43 health care personnel (HCP) who were exposed to a hospitalized patient with COVID-19, became symptomatic, and were tested for SARS-CoV-2 — Solano County, California, February 2020

Characteristic	No. (%)
**Total HCP**	**43 (100)**
**Age in yrs, median (range)**	39 (27–60)
**Sex**	
Female	36 (84)
Male	7 (16)
Risk category*	
High	5 (12)
Medium	36 (84)
Low	2 (5)
**Days from last contact with index patient to SARS-CoV-2 specimen collection, median (range)**	10 (8–14)
**Job title**	
Registered nurse	22 (51)
Respiratory therapist	4 (9)
Phlebotomist	4 (9)
Certified nursing assistant	3 (7)
Physician	3 (7)
Environmental services worker	3 (7)
Nutrition services worker	2 (5)
Pharmacist	1 (2)
Other	1 (2)

**Abbreviation:** COVID-19 = coronavirus disease 2019.

* According to initial risk stratification by hospital and public health staff members.

Among 43 HCP who were tested, 84% were female, 51% were registered nurses, and 95% were at high or medium risk (Table 1). Among the three HCP with COVID-19, two had high-risk and one had medium-risk exposures. Both HCP at high risk who developed COVID-19 had frequent, close contact with the index patient; one reported being present for a total of 3 hours while the patient was on BiPAP, and the other participated in BiPAP placement and intubation. Neither wore a facemask, respirator, eye protection, or gown. The third staff member with COVID-19, who was at medium risk, reported close contact with the patient for a total of 2 hours but not during AGPs. This staff member reported wearing a facemask and gloves most of the time but removed the mask occasionally to speak and did not wear eye protection.

Seventeen (46%) of 37 interviewed HCP reported exposure to the patient during at least one AGP (Table 2).** Being present for or assisting with nebulizer treatments was more common among HCP who developed COVID-19 (67%) than among those who did not (9%) (p = 0.04); being present for or assisting with BiPAP was also more common among HCP with COVID-19, although the difference was not statistically significant (p = 0.06). The median estimated duration of overall exposure to the patient was higher among HCP with COVID-19 (120 minutes) than among those without COVID-19 (25 minutes) (p = 0.06). Similarly, the median duration of exposure during AGPs^††^ was higher among HCP with COVID-19 (95 minutes) than among those without COVID-19 (0 minutes) (p = 0.13) (Table 3). Among non-AGP clinical activities, performing a physical examination was more common among HCP with COVID-19 (p = 0.02) (Table 2). Some HCP reported wearing gloves or facemasks during index patient care activities (Table 3); however, none reported use of eye protection, gowns, N95 respirators, or powered air-purifying respirators (PAPRs). At hospital B, 146 HCP had high-, medium-, or low-risk exposures; eight became symptomatic and were tested, none of whom had SARS-CoV-2 detected (CS Martin, MSN, personal communication, 2020).

**TABLE 2:** Reported patient care activities, including aerosol-generating procedures (AGPs), conducted by 37 health care personnel (HCP) who were tested for SARS-CoV-2 and participated in interviews — Solano County, California, February 2020

Exposures	No. (%)		p-value
	HCP with COVID-19	HCP without COVID-19	
**Total HCP**	**3**	**34**	**N/A**
Non-AGP activities*			
Taking vital signs	2 (67)	7 (21)	0.14
Taking medical history	1 (33)	7 (21)	0.53
Performing physical exam	3 (100)	8 (24)	0.02
Providing medication	1 (33)	10 (29)	1.00
Bathing or cleaning patient	0 (0)	4 (12)	1.00
Lifting or positioning patient	1 (33)	12 (35)	1.00
Emptying bedpan	1 (33)	2 (6)	0.23
Changing linens	0 (0)	5 (14)	1.00
Cleaning patient room	0 (0)	4 (12)	1.00
Peripheral line insertion	0 (0)	1 (3)	1.00
Central line insertion	0 (0)	1 (3)	1.00
Drawing arterial blood gas	1 (33)	1 (3)	0.16
Drawing blood	0 (0)	5 (15)	1.00
Manipulation of oxygen mask or tubing	2 (67)	5 (15)	0.09
Manipulation of ventilator or tubing	0 (0)	7 (21)	1.00
In room while high-flow oxygen being delivered	1 (33)	9 (26)	1.00
Collecting respiratory specimen	0 (0)	3 (9)	1.00
AGPs*^,†^			
Airway suctioning	0 (0)	7 (21)	1.00
Noninvasive ventilation (BiPAP, CPAP)	2 (67)	4 (12)	0.06
Manual (bag) ventilation	1 (33)	2 (6)	0.23
Nebulizer treatments	2 (67)	3 (9)	0.04
Breaking ventilation circuit	0 (0)	5 (15)	1.00
Sputum induction	0 (0)	1 (3)	1.00
Intubation	1 (33)	2 (6)	0.23
Performed or assisted	1 (33)	1 (3)	0.16
Present in room	0 (0)	1 (3)	1.00
Bronchoscopy	0 (0)	3 (9)	1.00
Performed or assisted	0 (0)	1 (3)	1.00
Present in room	0 (0)	3 (9)	1.00
**Any AGP**	**2 (67)**	**15 (44)**	**0.58**

**Abbreviations:** BiPAP = bilevel positive airway pressure; COVID-19 = coronavirus disease 2019; CPAP = continuous positive airway pressure; N/A = not applicable.

* Other patient care activities addressed in the exposure assessment tool but not listed here were not reported by any interviewed HCP.

^†^ For all AGPs listed here except intubation and bronchoscopy, exposure to AGP includes either performing or assisting with the procedure or being present in the patient’s room while the procedure was being performed. For intubation and bronchoscopy, performing or assisting with the procedure and being present in the room are presented separately.

**TABLE 3:** Reported personal protective equipment (PPE) use and exposure characteristics among 37 health care personnel (HCP) who were tested for SARS-CoV-2 and participated in interviews — Solano County, California, February 2020

Exposures	No./Total no. (%)		p-value
	HCP with COVID-19	HCP without COVID-19	
Reported always* using specified PPE during AGPs^†,§^ with index patient			
Gloves	2/2 (100)	10/16 (63)	0.53
Facemask	0/2 (0)	3/16 (19)	1.00
Reported always* using specified PPE during non-AGP activities^†^ with index patient			
Gloves	3/3 (100)	21/34 (62)	0.54
Facemask	0/3 (0)	3/34 (9)	1.00
**Duration of exposure to index patient**			
**Longest single duration of time in room (mins)**			
<2	0/3 (0)	2/34 (6)	0.70
2–30	2/3 (67)	23/34 (68)	
31–60	0/3 (0)	4/34 (12)	
>60	1/3 (33)	3/34 (9)	
**Median (IQR) total estimated time in patient room, mins**	120 (120–420)	25 (10–50)	0.06
**Median (IQR) total estimated time in patient room during AGPs, mins^¶^**	95 (0–160)	0 (0–3)	0.13
**Came within 6 ft of index patient**	3/3 (100)	30/34 (91)	1.00
**Reported direct skin-to-skin contact with index patient**	0/3 (0)	8/34 (24)	1.00
**Index patient either masked or on closed-system ventilator when contact occurred**			
Always	0/3 (0)	7/34 (23)	0.58
Sometimes	2/3 (67)	10/34 (32)	
Never	1/3 (33)	14/34 (45)	

**Abbreviations:** AGPs = aerosol-generating procedures; COVID-19 = coronavirus disease 2019; IQR = interquartile range.

* Versus sometimes or never.

^†^ No HCP reported use of gowns, N95 respirators, powered air-purifying respirators (PAPRs), or eye protection during any patient care activities for index patient.

^§^ Denominators for PPE use during AGPs are numbers of HCP exposed to AGPs.

**^¶^** This was estimated by asking each interviewed staff member to report the number and average duration of each exposure to the patient during AGPs. Total estimated duration for each AGP was calculated by multiplying the number of exposures by average duration of exposure during that AGP. Total estimated exposure time for all AGPs was calculated by adding total duration of exposures across all AGPs.

## Discussion

HCP are at high risk for acquiring infections during novel disease outbreaks, especially before transmission dynamics are fully characterized. The cases reported here are among the first known reports of occupational transmission of SARS-CoV-2 to HCP in the United States, although more cases have since been identified (*2*). Little is known to date about SARS-CoV-2 transmission in health care settings. Reports from Illinois, Singapore, and Hong Kong have described cohorts of HCP exposed to patients with COVID-19 without any documented HCP transmission (*3*–*5*); most HCP exposures in these cases occurred with patients while HCP were using contact, droplet, or airborne precautions.^§§^ As community transmission of COVID-19 increases, determining whether HCP infections are acquired in the workplace or in the community becomes more difficult. This investigation presented a unique opportunity to analyze exposures associated with COVID-19 transmission in a health care setting without recognized community exposures. Describing exposures among HCP who did and did not develop COVID-19 can inform guidance on how to best protect HCP.

Among a cohort of 121 exposed HCP, 43 of whom were symptomatic and tested, three developed confirmed COVID-19, despite multiple unprotected exposures among HCP. HCP who developed COVID-19 had longer durations of exposure to the index patient; exposures during nebulizer treatments and BiPAP were also more common among HCP who developed COVID-19. These findings underscore the heightened COVID-19 transmission risk associated with prolonged, unprotected patient contact and the importance of ensuring that HCP exposed to patients with confirmed or suspected COVID-19 are protected. CDC recommends use of N95 or higher-level respirators and airborne infection isolation rooms when performing AGPs for patients with suspected or confirmed COVID-19; for care that does not include AGPs, CDC recommends use of respirators where available.^¶¶^ In California, the Division of Occupational Safety and Health Aerosol Transmissible Diseases standard requires respirators for HCP exposed to potentially airborne pathogens such as SARS-CoV-2; PAPRs are required during AGPs.***

Studies of other respiratory pathogens have documented increased transmission risk associated with AGPs, many of which can generate large droplets as well as small particle aerosols (*6*). A recent study found that SARS-CoV-2 generated through nebulization can remain viable in aerosols <5 *μ*m for hours, suggesting that SARS-CoV-2 could be transmitted at least in part through small particle aerosols (*7*). Among the three HCP with COVID-19 at hospital A, two had index patient exposures during AGPs; one did not and reported wearing a facemask but no eye protection for most of the contact time with the patient. Given multiple unprotected exposures among HCP in this investigation, separating risks associated with specific procedures from those associated with duration of exposure and lack of recommended PPE is difficult. More research to determine the risks associated with specific procedures and the protectiveness of different types of PPE, as well as the extent of short-range aerosol transmission of SARS-CoV-2, is needed.

Patient source control (e.g., patient wearing a mask or connected to a closed-system ventilator during HCP exposures) might also reduce risk of SARS-CoV-2 transmission. Although the index patient was not masked or ventilated for the majority of hospital A admission, at hospital B, where the patient remained on a closed system ventilator from arrival to receiving a positive test result, none of the 146 HCP identified as exposed developed known COVID-19 infection (*8*). Source control strategies, such as masking of patients, visitors, and HCP, should be considered by health care facilities to reduce risk of SARS-CoV-2 transmission.

This findings in this report are subject to at least three limitations. First, exposures among HCP were self-reported and are subject to recall bias. Second, the low number of cases limits the ability to detect statistically significant differences in exposures and does not allow for multivariable analyses to adjust for potential confounding. Finally, additional infections might have occurred among asymptomatic exposed HCP who were not tested, or among HCP who were tested as a result of timing and limitations of nasopharyngeal and oropharyngeal specimen testing; serologic testing was not performed.

To protect HCP caring for patients with suspected or confirmed COVID-19, health care facilities should continue to follow CDC, state, and local infection control and PPE guidance. Early recognition and prompt isolation, including source control, for patients with possible infection can help minimize unprotected and high-risk HCP exposures. These measures are crucial to protect HCP and preserve the health care workforce in the face of an outbreak already straining the U.S. health care system.

**Summary**

**What is already known about this topic?**

Health care personnel (HCP) are at heightened risk of acquiring COVID-19 infection, but limited information exists about transmission in health care settings.

**What is added by this report?**

Among 121 HCP exposed to a patient with unrecognized COVID-19, 43 became symptomatic and were tested for SARS-CoV-2, of whom three had positive test results; all three had unprotected patient contact. Exposures while performing physical examinations or during nebulizer treatments were more common among HCP with COVID-19.

**What are the implications for public health practice?**

Unprotected, prolonged patient contact, as well as certain exposures, including some aerosol-generating procedures, were associated with SARS-CoV-2 infection in HCP. Early recognition and isolation of patients with possible infection and recommended PPE use can help minimize unprotected, high-risk HCP exposures and protect the health care workforce.

## Footnotes

* Exposure was defined according to CDC guidance for HCP with potential exposure to COVID-19, which categorizes exposures based on factors such as exposure to the patient during AGPs, personal protective equipment use, and source control (e.g., patient wearing a facemask) during exposure. https://www.cdc.gov/coronavirus/2019-ncov/hcp/guidance-risk-assesment-hcp.html.

† HCP categorized as being at low risk were allowed to continue to report to work but were checked for symptoms before the start of each shift; no additional follow-up was conducted for HCP categorized as having no identifiable risk.

§ Including fever (subjective or measured at ≥100.4°F [38°C]), cough, shortness of breath, or sore throat.

¶ One of the remaining six HCP declined to participate; the other five could not be reached after at least three attempted phone calls.

** For the purposes of this report, the following procedures during the patient’s hospitalization were considered AGPs: airway suctioning, noninvasive positive pressure ventilation including BiPAP, manual ventilation, nebulizer treatments, breaking the ventilator circuit, sputum induction, intubation, and bronchoscopy. Exposure during an AGP included both direct participation in AGP (i.e., performing or assisting with intubation), as well as presence in the patient’s room while AGP was being performed.

†† This was estimated by asking interviewed HCP to report the number and average duration of exposures to the patient during each AGP. Total estimated duration for each AGP was calculated by multiplying the number of exposures by average duration of exposure during that AGP. Total estimated exposure time for all AGPs was calculated by adding total duration of exposures across all AGPs.

§§ Additional detail on recommended transmission-based precautions recommended for patients with suspected or confirmed COVID-19. https://www.cdc.gov/coronavirus/2019-ncov/infection-control/control-recommendations.html.

¶¶ https://www.cdc.gov/coronavirus/2019-ncov/infection-control/control-recommendations.html.

*** Aerosol Transmissible Diseases. California Code of Regulations, Section 5199 (2009). https://www.dir.ca.gov/title8/5199.html.
